# Profile of Microbial Keratitis

**DOI:** 10.7759/cureus.20663

**Published:** 2021-12-24

**Authors:** James Lim Wen Siang, Ong Wu Zhuan, Ngio Yi Chen, Ng Sok Lin

**Affiliations:** 1 Ophthalmology, Taiping Hospital, Perak, MYS

**Keywords:** district hospital, retrospective study, pseudomonas, microbial keratitis, corneal ulcer

## Abstract

Purpose: To study the demographic characteristics, predisposing factors, and latest trends of causative organisms and to analyze the prognostic factors of visual outcome in microbial keratitis.

Methods: A retrospective study of patients diagnosed with microbial keratitis who required hospital admission in the period between January 2018 and December 2020 in Taiping Hospital, Perak, Malaysia.

Results: A total of 75 eyes of 74 patients who were admitted to the hospital were studied. The male to female ratio was 13.8:1. Seventy percent of patients in this study were within the productive age group between 20 and 59 years old, with a mean age of 48 years old, and 51.4% of them were labourers. Cornea foreign bodies (42, 56%) were the most common predisposing factors and were associated with good visual outcomes (P<0.005). Other significant predictors for the final visual outcome were: presenting visual acuity, size of ulcer, duration of hospitalization, and duration of resolution. The mean duration of hospitalization was seven days. Corneal scrapings were done in all cases where 44 eyes (58.7%) were found to be positive for growth. Ten eyes (13.3%) that ended up with evisceration yielded a positive result. Gram-negative bacteria was the most prevalent causative organism of infective keratitis in the local/this region. Pseudomonas sp (20, 26.7%) being the most common bacterial isolate, was seen in all four contact lens-related cases and was associated with poor visual outcome and a high rate of evisceration. Patients who developed complications such as cornea melting (9, 12%), cornea perforation (11, 14.7%) and endophthalmitis (7, 9.3%) were associated with poor visual outcomes. Likewise, patients who required therapeutic interventions such as corneal gluing, tarsorrhaphy, and penetrating keratoplasty generally had poor visual outcomes (P<0.005; P=0.000008).

Conclusion: Microbial keratitis is a major cause of ocular morbidity globally. Understanding the demographic and epidemiological characteristics of microbial keratitis of the region is important in the initial prompt treatment of the patients and may eventually improve the visual outcome.

## Introduction

Microbial keratitis, which is also known as corneal ulcer, is a major cause of visual impairment and blindness globally [1]. It can lead to significant visual impairment, which includes corneal scarring, vascularization, or corneal perforations that warrant evisceration or enucleation [2], adding to the social and healthcare burden of the individual and the community [3]. Important risk factors include corneal trauma, contact lens wear, previous ocular surgery, and ocular surface disease. The incidence of different microorganisms and their antibiotic resistance patterns varies worldwide and changes over time, hence the need to review the standard protocols for managing microbial keratitis regularly [3].

The objective of this study was to ascertain the socio-demography, predisposing factors, latest trend of causative organism, treatment given, complications, and visual outcome of microbial keratitis. Besides this, it was to determine the association between presenting visual acuity, final visual acuity, risk factor, and duration of hospital stay. Identification of these factors will help in providing a cost-effective approach to the diagnosis, management, and prevention of this condition [4].

## Materials and methods

This was a retrospective study of patients diagnosed with microbial keratitis who required hospital admission in the period between January 2018 and December 2020 in Taiping Hospital, Perak, Malaysia. Taiping hospital is located in the northern region of Perak. Perak, with a population of 2.51 million, is one of the states in Malaysia. Being the second largest hospital in Perak, it is an important referral center for other district hospitals in northern Perak that cover approximately 1/3 of the state population.

The relevant data were traced and acquired from the hospital’s medical records unit. Data collection was done to include the demographic data (age, race, gender, and occupation) and clinical characteristics of the patients, which included the predispose factor, presenting visual acuity, presentation interval, ocular examination findings (size of corneal ulcer, location, presence of hypopyon, corneal melting, and high intraocular pressure [IOP]), complications (if any), any surgical intervention (if any), length of hospital admission, and final visual acuity. Corneal scraping results (Gram stain, fungal stain, and culture) and treatment received were also analyzed.

Corneal scrapes were obtained with a sterile needle for Gram stain and fungal stains (potassium hydroxide preparation). Corneal smears were obtained with a sterile needle for in vitro culture. In vitro culture included chocolate agar, blood agar, and Sabouraud’s dextrose agar plates.

Local broad-spectrum antibiotics (ceftazidime 5%, fortified gentamicin 0.9%), antifungals (fluconazole 0.9%, Amphotericin B 0.125%), and cycloplegic eye drops were ordered for every patient on first presentation at our center. Systemic antibiotics (doxycyline/ciprofloxacin, or both of them) and systemic antifungals (fluconazole) will be ordered depending on the individual case. Treatment will then be modified if indicated by culture results, antibiotic sensitivity, and clinical response.

The study population includes all patients (including both male and female patients) who were diagnosed with microbial keratitis and who required hospital admission in the period between January 2018 and December 2020 in Taiping Hospital, Perak, Malaysia.

Patients whose age ranged from 18 to 85 years old with microbial keratitis who required hospital admission within the period of January 2018 until December 2020 were included in the study.

Patients who were treated as outpatients, defaulted on follow-up within less than four weeks after first presentation, vernal keratoconjunctivitis, and viral keratitis were excluded.

Data analysis was performed using the Statistical Package for Social Sciences (SPSS) version 26.0 (IBM Corp., Armonk, NY). Descriptive statistics were presented as a mean (standard deviation), and categorical data as numerical variables and frequency (percentage). The Chi square, or Fisher’s exact test, was used to determine the association between categorical variables. The Mc Nemar chi-square test was performed to determine the change in vision between pre and post treatment. A P-value of <0.05 was considered statistically significant.

## Results

Demographics

A total of 74 patients with microbial keratitis who required hospital admission were identified from the patient database at Taiping Hospital. Of the 74 patients, 69 (93.2%) were males and 5 (6.8%) were females; the male to female ratio was 13.8:1. In Table 1, the distribution of cases according to age group is shown. The ages ranged from 15 to 85 years old, with a mean of 47.95 years old. Most of the patients predominantly fall into the age group of 40-59 years old, and most of them were Malay (78.4%) and labourers (51.4%), as shown in Tables 2 and 3, respectively.

**Table 1:** Age group and gender distributions

Age group (years)	Male, n (%)	Female, n (%)
0-19	2 (2.70)	0 (0)
20-39	20 (27.0)	2 (2.7)
40-59	32 (43.2)	1 (1.4)
60-79	14 (18.9)	1 (1.4)
80 and above	1 (1.4)	1 (1.4)
Total	69 (93.2)	5 (6.8%)

**Table 2:** Race distributions

Race	Number (%)
Malay	58 (78.4)
Indian	8 (10.8)
Chinese	6 (8.1)
Other	2 (2.7)

**Table 3:** Occupation distributions

Occupation	Number (%)
Labourer	38 (51.4)
Office worker	3 (4.1)
Student	2 (2.7)
Unemployed	11 (14.9)
Other	20 (27.0)

A total of 75 eyes from 74 patients were included in this study. Forty-six cases (62.16%) involved the right eye, 27 cases (36.49%) involved the left eye, and 1 patient (1.35%) had bilateral eye involvement. Most of them (n=45, 60%) presented within up to three days following the onset of the symptoms, as shown in Table 4.

**Table 4:** Clinical characteristic and association factors for the final visual acuity *Final visual acuity of ≥6/60 included total 10 post eviscerated eyes.

Clinical characteristic	Number (%)	Final visual acuity		
		<6/60	≥6/60*	
Presenting visual acuity				
<6/60	37 (49.33)	30	7	P<0.05
≥6/60	38 (50.67)	14	24	
Presentation interval				
<4 days	45 (60.0)	30	15	P<0.085
≥4 days	30 (40.0)	14	16	
Location of ulcer				
Central	40 (53.3)			
Paracentral	26 (34.7)			
Peripheral	9 (12.0)			
Size of ulcer				
≤4 mm	42 (56.0)	34	8	P<0.05
>4 mm	33 (44.0)	10	23	
Hypopyon				
Present	34 (45.33)	17	17	P=0.165
Absent	41 (54.67)	27	14	
High intraocular pressure				
Yes	37 (49.33)	19	18	P=0.204
No	38 (50.67)	25	13	
Duration of hospitalization				
≤7 days	44 (58.67)	31	13	P<0.05
>7 days	31 (41.33)	13	18	
Duration of resolution				
≤6 weeks	30 (40.0)	22	8	P<0.05
>6 weeks	45 (60.0)	22	23	
Final visual acuity*				
<6/60	44 (58.7)			
≥6/60	31 (41.3)			

Predisposing factors

Ocular trauma was the most common predisposing factor (60%) for microbial keratitis. Ocular trauma was further divided into corneal foreign bodies (42 eyes) and periocular trauma (3 eyes). Of these, 26 eyes (34.67%) were caused by organic matter and 16 eyes (21.33%) were caused by non-organic matter. Three eyes had recurrent corneal ulcers in post-penetrating keratoplasty eyes, while another six eyes had underlying ocular surface disease, and four others were contact lens related. Seventeen (22.67%) of the cases had no identifiable predisposing factor (Table 5).

**Table 5:** Predisposing factors

Predisposing factors	Number (%)
Trauma	
· Corneal foreign body	
Organic	26 (34.67)
Inorganic	16 (21.33)
· Periocular trauma	3 (4.0)
Recurrent corneal ulcer	3 (4.0)
Ocular surface disease	6 (8.0)
Contact lens	4 (5.33)
Unknown	17 (22.67)

Clinical characteristics and hospitalization

Of the 74 studied patients, 10 (13.5%) had diabetes mellitus. The relevant clinical signs of the studied cases, such as initial and final visual acuity, location and size of the corneal ulcer, presence of hypopyon, presence of high intraocular pressure, as well as cornea melting and cornea perforation, were studied. Their associations with the final visual outcome were analyzed and are shown in Table 5. The duration of hospitalization ranged from 1 day to 26 days, with a mean of seven days. Presenting visual acuity, size of ulcer, duration of hospitalization and resolution time were found to be significantly associated with the final visual acuity (p<0.05). Otherwise, the presentation interval, presence of hypopyon, and high intraocular pressure were not associated with the final visual acuity.

More than half (58.7%) of the studied eyes had achieved good final visual acuity (<6/60) in this study. Cornea foreign body as the most common predisposing factor was associated with good final visual acuity as shown in Table 6.

**Table 6:** Clinical characteristic and association factors for the final visual acuity

Predispose factor	Number (%)	Final visual acuity		
		<6/60	≥6/60	
Cornea foreign body				
Yes	42 (56)	31	11	P<0.05
No	33 (44)	13	20	

Microbiological profile

All the patients were investigated for corneal tissue culture and sensitivity. Only the organisms isolated from the first corneal tissue culture at the initial presentation were taken into account. Forty-four (58.67%) of the 75 eyes were cultured positive, and 31 (41.33%) of the eyes revealed no growth. Organisms isolated from the culture positive group are shown in Table 7. In 4 out of the 44 culture positive cases (9.1%), more than one organism was isolated, i.e., *Enterobacter cloacae* with *Klebsiella ozaenae*, *Pseudomonas aeruginosa*, *Streptococcus pneumoniae* with *Staphylococcus aureus*, as well as two cases of mixed *P. aeruginosa* and fungal infection. In this study, *P. aeruginosa* was identified as the most common causative organism for microbial keratitis, followed by *S. pneumoniae* (Table 8).

**Table 7:** Association of Pseudomonas aeruginosa with final visual acuity and evisceration

Causative organism	Number (%)	Final visual acuity			Evisceration done (n)	
		<6/60	≥6/60		Yes	No
P. aeruginosa						
Yes	20 (26.67)	8	12	P<0.05	6	14
No	55 (73.33)	36	19		4	51

**Table 8:** Isolated organisms by culture *Polymicrobial cultures. ^#^Percentage of culture positive cases(n=44). Total is greater than 100% because of polymicrobial infections.

Organisms	Number of isolates* (n=48)	Percentage^#^ (n=44)
Total bacteria	38	86.4
Total Gram positive	10	22.7
*Streptococcus pneumoniae*	5	11.4
*Staphylococcus epidermidis*	2	4.5
*Staphylococcus aureus*	2	4.5
*Streptococcus agalactiae*	1	2.3
Total Gram negative	28	63.6
*Pseudomonas aeruginosa*	19	43.2
*Klebsiella aerogenes*	2	4.5
*Serratia marcescens*	2	4.5
*Enterobacter cloacae*	2	4.5
*Acinetobacter ursingii*	1	2.3
*Klebsiella ozaenae*	1	2.3
*Klebsiella pneumoniae*	1	2.3
Fungus	10	22.7
Fusarium sp	1	2.3
*Fusarium solani*	1	2.3
Arthrographis spp	1	2.3
Acremonium spp	1	2.3
Non sporulating mould	1	2.3
Penicillium sp	1	2.3
Mix growth of fungus (unable to identify)	4	9.1

Among the 10 (13.33%) eyes that required evisceration, 100% of them were culture positive. Six (60%) of them were caused by *P. aeruginosa*, 1 (10%) *K. pneumoniae*, 1 (10%) *S. agalactiae*, 1 (10%) *Acinetobacter ursingii*, and 1 (10%) case of mixed infections with *S. pneumoniae* and *S. aureus*.

In cases where *P. aeruginosa* was isolated, they were significantly associated with poor visual outcome and evisceration (Table 8). Contact lens-related microbial keratitis was also significantly associated with *P. aeruginosa* with a P=0.004. All four (100%) cases of contact lens-related microbial keratitis were related to *P. aeruginosa*.

Complications

Serious ocular complications secondary to infective keratitis include cornea melting, cornea perforation, and endophthalmitis. They were all significantly related to the poor visual outcome (Table 9).

**Table 9:** Complications and association with the final visual acuity

Complications	Number (%)	Final visual acuity		
		<6/60	≥6/60	
Cornea melting				
Present	9 (12.0)	1	8	P<0.05
Absent	66 (88.0)	43	23	
Cornea perforation				
Yes	11 (14.67)	1	10	P<0.05
No	64 (85.33)	43	21	
Endophthalmitis				
Yes	7 (9.33)	0	7	P<0.05
No	68 (90.67)	44	24	

Treatment

The first line of treatment included empirical topical antibiotics with and without topical antifungals. In more severe cases, systemic antibiotics and systemic antifungal coverage were added.

Topical antibiotics that were used in our cases included fortified gentamicin 0.9%, ceftazidime 0.5%, and moxifloxacin 0.5%, whereas topical anti-fungals used included Amphotericin B 0.125% and Fluconazole 0.2%. In this study, a total of 33 (44.0%) patients were empirically covered with two topical antibiotics and two topical antifungals. Forty (53.33%) patients started with only topical antibiotics and six (8.0%) patients started with only topical anti-fungals. A total of 29 (38.67%) cases received systemic antibiotics and 26 (34.67%) cases received systemic anti-fungals throughout the treatment.

As complications arose, four (5.33%) patients required cornea gluing, two (2.67%) required tarsorrhaphy, two (2.67%) required penetrating keratoplasty, and 10 (13.33%) ended up with evisceration. One patient had cornea gluing done before proceeding with penetrating keratoplasty. Generally, the patients who needed therapeutic interventions had poor visual outcomes (Table 10).

**Table 10:** Therapeutic interventions done correlating with the final visual acuity *Vision not available (post-evisceration). ^#^Therapeutic interventions included corneal gluing, tarsorrhaphy, penetrating keratoplasty, and evisceration.

Therapeutic interventions	Number (%)	Final visual acuity		
		<6/60	≥6/60	
Corneal gluing				
Yes	4 (5.33)	1	3	P=0.3
No	71 (94.67)	43	28	
Tarsorrhaphy				
Yes	2 (2.67)	0	2	P=0.168
No	73 (97.33)	44	29	
Penetrating keratoplasty				
Yes	2 (2.67)	1	1	P=1.0
No	73 (97.33)	43	30	
Evisceration				
Yes	10 (13.33)	0	10*	P<0.05
No	65 (86.67)	44	21	
Therapeutic interventions^#^				
Yes	17 (22.67)	2	15	P<0.05
No	58 (77.33)	42	16	

## Discussion

Socio-demography and the predisposing risk factors for microbial keratitis were found to be variable in different regions, populations, and geographical locations [3]. With time, these microbial profiles may change too [5]. The mainstay treatment for microbial keratitis is topical antibiotics, which are started empirically and then changed based on the clinical response and sensitivity results of the isolated organism [6].

Our study has shown that the male gender was more commonly affected, and this is in line with other studies conducted among Asian countries [4-7], compared to those of Western countries [3,8,9]. It occurs most commonly within the productive age group [7]. Seventy percent of patients in this study were within the age group of 20-59 years old, and 51.4% of the study subjects were labourers. This can be correlated to the socio-economic status of individuals in the study depending on their occupation [7].

In our study, ocular trauma (mainly foreign bodies in the cornea) was reported to be the main predisposing factor of microbial keratitis, which is supported by another study by Kursiah et al. [6]. The presence of manufacturing and construction industries in the nearby areas of our study may explain why trauma is the most frequent risk factor for microbial keratitis in our study. In Malaysia, trauma was the main predisposing factor in sub-urban regions [10] and contact lenses in the more urbanized areas [5,11]. Ocular surface disease was found to be the most common predisposing factor as stated in Otri et al. [3], a study conducted in Nottingham, UK, where contact lenses were the most common risk factor stated in Puig et al. [8], a study conducted in Texas, USA. This supported the theory that predisposing risk factors for microbial keratitis are variable in different populations and geographical locations [3,5].

In our study, more than half (60%) of the patients presented within three days or less after the onset of symptoms. This is similar to the findings in Omar et al. where the mean presenting time was 4.67 days [5] but contrasts with the longer presentation time, which was 8.9 days and 21.3 days reported in Wong et al. [12] and Toth et al. [9], respectively. The differences could be due to cultural issues, financial status, awareness or access to eye care facilities [3,5]. Taiping hospital was readily accessible and this may contribute to the earlier presentation after symptoms onset.

According to some studies, the size of the ulcer, but not the hypopyon, is related to poorer visual outcomes [5,11]. Our study had similar findings. More than half (56%) of our cases had size of infiltrate ≤4 mm and this could be due to shorter interval of presentation [5]. We also found that the smaller size of the infiltrate was associated with better visual prognosis. A higher proportion of central keratitis was found in this study, which is similar to Omar et al. [5], which reported that large and centrally located keratitis were associated with hypopyon.

In our study, 49.33% of patients had secondary raised IOP, but their association with poor visual prognosis was not statistically significant. Nevertheless, special attention should also be given to intraocular pressure as secondary raised IOP can lead to more complications such as corneal perforation and further visual loss [3].

The rate of cultural positivity in this study was 58.7%, which was higher than the studies by Otri et al. in the United Kingdoms (41%) [3], Omar et al. in Malaysian urban areas (47.5%) [5], and Thailand (25.6-30.16%) [13], but lower than the studies in the United States (63-82%) [14] and New Zealand (71%) [12]. Corneal scraping technique, methods of culturing, types of causative organism [5], different types of culturing medium, and antibiotic treatment prior to corneal scraping [3] could be the reasons contributing to this variation. Using the correct medium for culture, proper scraping technique, and handling of the specimen could improve the culture result [3], as a positive culture enables a sensitivity test and increases the opportunity to control the infection [5].

Similar to other studies, most of the microbial keratitis cases were due to Gram-positive organisms [3,6,8,9,15]. Toth et al. and Puig et al. stated that CoNS (coagulase-negative staphylococci) were the most frequently isolated bacteria. However, as a major component of the normal flora of human skin and conjunctiva, CoNS are primarily regarded as contaminants with a low virulence factor [8,9]. In contrast to our study and other local Malaysian studies [6,7], P. aeruginosa was found to be the main causative organism along with other Gram-negative bacteria. Pseudomonas was the most common organism responsible for contact lens-related microbial keratitis [3,6,8]. It was isolated in all cases of contact lens-induced keratitis in our study. In our study, P. aeruginosa microbial keratitis was significantly associated with poor visual prognosis and a high rate of evisceration. It can cause complete corneal melting within short period of time, therefore prompt diagnosis and early aggressive treatment is essential in case of microbial keratitis caused by Pseudomonas [3,9].

Duration of hospitalization and duration of resolution were significantly associated with the final visual outcome found in our study. The duration of hospital stay and treatment can be quite variable depending on the strain of the causative organism [3,6] and the patient’s general and ocular health [3]. Some resistant strain had been identified and longer duration of treatment is needed to eradicate the infection thence prolonging the hospital stay [6].

Increased age was found to be one of the risk factors for microbial keratitis-related endophthalmitis and loss of the eye [16]. In our study, there were seven (9.3%) patients complicated by endophthalmitis, which was associated with a poor visual outcome. A total of 10 (13.3%) patients underwent evisceration due to perforated corneas. In Hungary, the most common indications for enucleation and evisceration were non-resolving keratitis in 62.5% and severe keratitis with endophthalmitis in 37.5% of the sample [9], but in Australia, only 4.3% of the patients were associated with endophthalmitis [17].

The limitations of this study include that it was performed in a retrospective way and Taiping Hospital is still using a manual filing system for patients’ medical records. There were difficulties in tracing the medical records, some data were missing, and the medical records are only kept for three years, all of which limited the study's sample size. A larger prospective multi-center researches should be conducted to gather more data and sample size, and eventually improve the management of microbial keratitis in future.

## Conclusions

Males in the productive age group who worked as labourers were prone to sight-threatening microbial keratitis. Ocular trauma, especially corneal foreign bodies, was the most common predisposing factor. This highlights the need to emphasize safety precautions and protective eye gear usage when dealing with high-risk work. Early presentation and early empirical treatment will definitely improve the visual outcome. In view of the changing and different trends of microbial keratitis in different geographical areas, an updated and comprehensive study according to the different regions is needed to guide the antimicrobial regime.
